# Different lasers in the treatment of benign prostatic hyperplasia: a network meta-analysis

**DOI:** 10.1038/srep23503

**Published:** 2016-03-24

**Authors:** Xingming Zhang, Pengfei Shen, Qiying He, Xiaoxue Yin, Zhibin Chen, Haojun Gui, Kunpeng Shu, Qidun Tang, Yaojing Yang, Xiuyi Pan, Jia Wang, Ni Chen, Hao Zeng

**Affiliations:** 1 Department of Urology, Institute of Urology, West China Hospital, Sichuan University, Chengdu, China, 610041; 2 Department of Pathology, West China Hospital, Sichuan University, Chengdu, China, 610041

## Abstract

All available surgical treatments for benign prostatic hyperplasia (BPH) have their individual advantages or disadvantages. However, the lack of head-to-head studies comparing different surgeries makes it unavailable to conduct direct analysis. To compare the efficacy and safety among different lasers and transurethral resection of prostate (TURP) for BPH, randomized controlled trials were searched in MEDLINE, EMBASE, Cochrane library, WHO International Clinical Trial Registration Platform, and Clinical Trial.gov by 2015.5; and the effectiveness-, perioperation- and complication-related outcomes were assessed by network meta-analysis. 36 studies involving 3831 patients were included. Holmium laser through resection and enucleation had the best efficacy in maximum flow rate. Thulium laser through vapo-resection was superior in improving international prostate symptom score and holmium laser through enucleation was the best for post-voiding residual volume improvement. Diode laser through vaporization was the rapidest in removing postoperative indwelling catheter, while TURP was the longest. TURP required the longest hospitalization and thulium laser through vapo-resection was relatively shorter. Holmium and thulium lasers seem to be relatively better in surgical efficacy and safety, so that these two lasers might be preferred in selection of optimal laser surgery. Actually, more large-scale and high quality head-to-head RCTs are suggested to validate the conclusions.

Benign prostatic hyperplasia (BPH) is one of the most important causes of lower urinary tract symptoms (LUTS) in men, especially the elder men. Although BPH is not considered as a life-threatening disease, its impact on patients’ quality of life should not be underestimated^1^. Treatments for BPH include watchful waiting, drug therapy and surgery. Although a majority of patients with BPH could be treated with watchful waiting or drug therapies (alpha-blockers, 5-alpha-reductase inhibitors, anticholinergics, phytotherapeutics alone or combinations), there is still a certain number of patients finally required surgical intervention, such as transurethral resection of prostate (TURP) and surgeries lasers^2^.

Even though TURP is still frequently used as traditional surgical therapy for BPH, in the past two decades, several novel lasers including holmium laser, thulium laser, KTP/Nd:YAG laser, Nd:YAG laser, diode laser and green light laser, have also shown excellent clinical effectiveness for BPH. All these available surgical treatments have their individual advantages or disadvantages. Abundant options faced by surgeons and patients result in the question that which treatment is relatively the best choice for BPH. However, because it is lack of head-to-head comparisons among different surgeries and the information about their comparative effectiveness is limited, direct statistical analysis is not available. Fortunately, a novel analysis method, network meta-analysis, might allow us to conduct a systematic review to compare the efficacy and safety among different surgical treatments for BPH.

## Results

### Study characteristics

**Figure 1:**
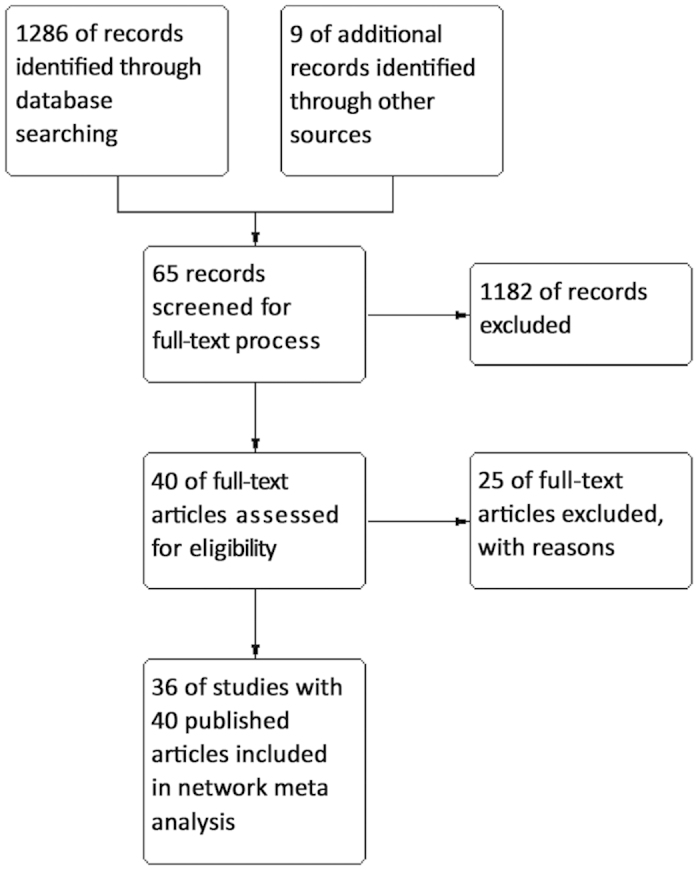
Flow diagram of search in the scientific literature to identify randomized controlled trials.

**Figure 2:**
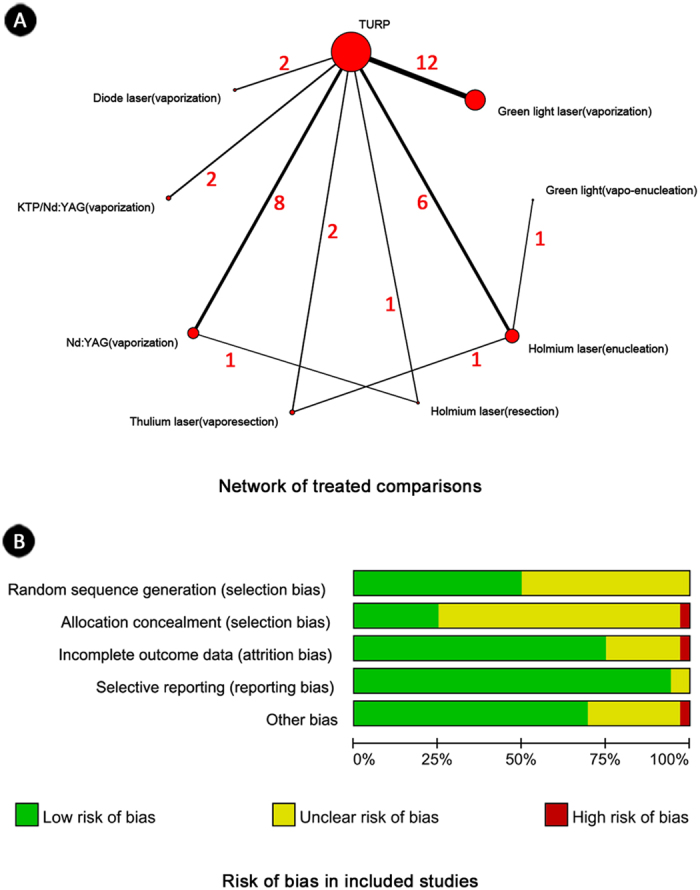
Network of treated comparisonsand risk of bias in included studies. (**A**) TURP was the most common comparison, and direct comparisons among laser techniques were less. Numbers beside the lines were the amounts of studies among comparisons. Degree of thickness of line also indicated quantity of studies among comparisons. (**B**) Five items introduced by Cochrane Hanbook were considered. Blinding was canceled due to impractical implementation.

1286 studies were identified through electronic searches and nine studies through additional searching. Thirty-six studies with 40 published articles (3831 participants) were finally included in our study^3^,^4^,^5^,^6^,^7^,^8^,^9^,^10^,^11^,^12^,^13^,^14^,^15^,^16^,^17^,^18^,^19^,^20^,^21^,^22^,^23^,^24^,^25^,^26^,^27^,^28^,^29^,^30^,^31^,^32^,^33^,^34^,^35^,^36^,^37^,^38^,^39^,^40^,^41^,^42^ (Fig. 1). The characteristics of the included studies were summarized in Table 1. Efficacy and safety of lasers were compared with TURP in 33 studies. Only three studies compared between different lasers (Fig. 2A).

Among reported lasers, Nd:YAG was the earliest laser used in the treatment of BPH^3^. Almost all studies investigated laser as monotherapy compared with standard treatment (TURP), and only two studies applied combined strategy—KTP plus Nd:YAG. Green light laser technique was the most commonly used technology and was reported in 13 studies, and seven studies exactly recorded the wavelength (532 nm). Studies varied in ways of treatment, surgical techniques and publication years (from 1995 to 2015); however, the baseline characteristics of all patients were basically similar among interventions. The main surgical technique of lasers was vaporization; enucleation and resection were mainly conducted by Holmium laser. One study stopped early due to the need for prolonged catheterization and a high rate of urinary tract infection^4^.

Risk of bias in included studies was summarized graphically in Fig.2B. One study had high risk of bias for sequence allocation and concealment, as it was reported as an open-label clinical trial^5^. Another trial was considered with high risk of other bias due to early discontinuation of study^4^. Since it was sometimes difficult to blind surgeons and patients, we did not include the blinding items of risk of bias in our analysis.

### Effectiveness-related outcomes

**Figure 3:**
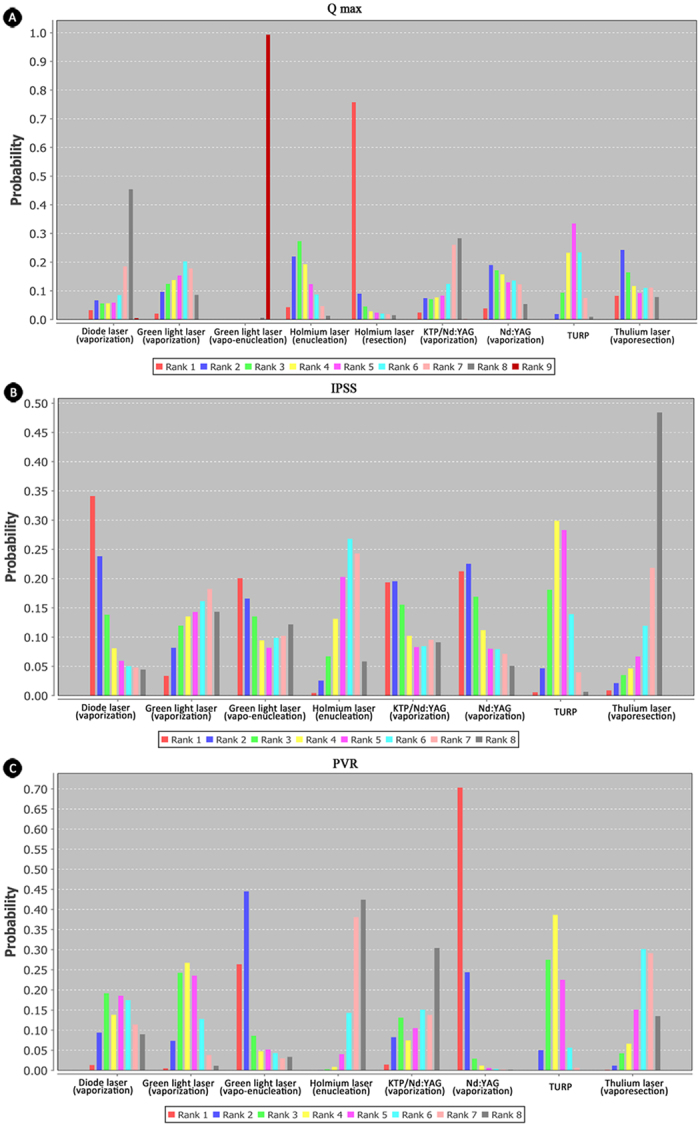
Rank of probability for effective outcomes. (**A**) rank probability of Q max; (**B**) rank probability of IPSS; (**C**) rank probability of PVR. All six kinds of lasers through different surgical techniques and TURP were availably compared in the network meta-analysis including Q max, IPSS and PVR. Q max-maximum flow rate. IPSS-International Prostate Symptom Score. PVR-Post-Void Residual.

Three effectiveness-related outcomes, including Q max, IPSS and PVR, were analyzed in the study. Based on node-split analyses, no significant inconsistencies were observed in effectiveness-related outcomes (p > 0.5). Q max was reported in 24 studies, involving seven interventions—green light, holmium laser, thulium laser, diode laser, Nd:YAG, KTP/Nd:YAG and TURP through different surgical techniques. Among them, holmium laser (resection and enucleation) was significantly superior to other lasers and TURP in improving Q max, and green light laser through vapo-enucleation was the worst one technique. The relative effect estimate of holmium laser (vaporization) versus green light laser (vapo-enucleation) was 16.69 (8.78, 25.00). Rank probability of Q-max (from best to worst) among lasers was holmium laser (resection)>holmium laser (enucleation)>thulium laser (vapo-resection)>Nd:YAG (vaporization)>TURP>green light (vaporization)>diode laser (vaporization)>green light (vapo-enucleation). (Fig. 3A)

In terms of IPSS score, thulium laser through vapo-resection showed significant superiority in improving IPSS over other interventions, rank probability (from best to worst): thulium laser (vapo-resection)>holmium laser (enucleation)>green light laser (vaporization)>TURP>green light laser (vapo-enucleation)>KTP/Nd:YAG (vaporization)>Nd:YAG (vaporization)>diode laser (vaporization) (Fig. 3B). For PVR, holmium laser through enucleation was the best technique and Nd:YAG (vaporization) was the worst one. The relative effect estimate was 40.36 (12.06, 71.23). Rank probability (from best to worst): holmium laser (enucleation)>thulium laser (vapo-resection)>KTP/Nd:YAG (vaporization)>diode laser (vaporization)>green light laser (vaporization)>TURP>green light laser (vapo-enucleation)>Nd:YAG (vaporization)(Fig. 3C).

### Perioperation-related outcomes

**Figure 4:**
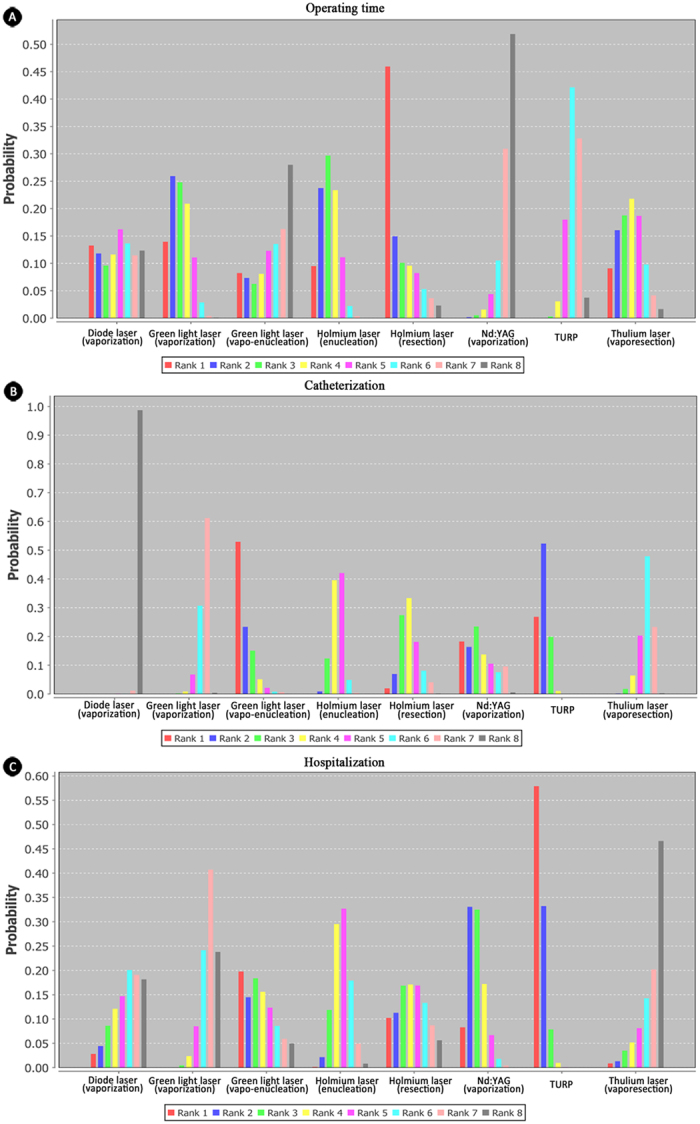
Rank of probability for perioperation-related outcomes. (**A**) rank probability of operating time; (**B**) rank probability of catheterization; (**C**) rank probability of hospitalization. Five kinds of lasers through different surgical techniquesand TURP were included for network meta-analysis about perioperation-related outcomes. Nd:YAG/KTP laser was not available.

Perioperation-related outcomes included operating time, duration of catheterization and stay of hospital. Consistency model was used for all perioperation-related outcomes. Twenty-five studies investigated operating time of lasers and TURP in the treatment of BPH. For operating time, seven interventions through different surgical techniques were availably compared—holmium laser, thulium laser, green light laser, Nd:YAG, diode laser and TURP. Nd:YAG laser through vaporization and TURP had much fewer operating time than others, while holmium laser (resection) took the longest time; the relative effect estimates of Nd:YAG laser (vaporization) and TURP versus holmium laser (resection) were −22.3 (−48.87, 4.37) and −11.45 (−21.04, −2.04), respectively. Rank probability: (from shortest to longest) Nd:YAG (vaporization)>TURP>diode laser (vaporization)>green light laser (vaporization)>thulium laser (vaporesection)>green light laser (vaporization)>holmium laser (enucleation)>holmium laser (resection) (Fig. 4A).

For catheterization, patients underwent diode laser (vaporization) were the rapidest technique in removing catheter after surgery, and TURP was the longest one; the relative effect estimate of TURP vs diode laser was 68.90 (47.35, 90.84). Rank probability from short to long: diode laser(vaporization)>green light laser(vaporization)>thulium laser(vaporesection)>holmium laser(enucleation)>holmium laser(resection)>TURP>Nd:YAG(vaporization)>green light laser(vapo-enucleation) (Fig. 4B). In terms of hospitalization, TURP required the longest time of stay in hospital, while thulium laser was relatively shortest, relative effect estimate was 46.56 (7.18, 86.27). Rank probability(from shortest to longest),: thulium laser (vapo-resection)>green light laser (vaporization)>diode laser (vaporization)>holmium laser (enucleation)>holmium laser (resection)>green light laser (vapo-enucleation)>Nd:YAG (vaporization)>TURP (Fig. 4C).

### Short-term complications-related outcomes

Short-term complications-related outcomes, including dysuria, urinary retention, re-catheterization, clot retention, transfusion, incontinence, TURS, and UTI were analyzed. Total of nine studies reported dysuria, which was availably compared in five interventions through different techniques (holmium laser, green light laser, KTP/Nd:YAG, Nd:YAG laser and TURP).The network analysis showed that dysuria was the most common short-term complication in patients who underwent green light laser(vapo-enucleation) and holmium laser(enucleation), and the least common in Nd:YAG laser with vaporization. The relative effect estimates of Nd:YAG(vaporization) versus green light laser (vapo-enucleation) and holmium (enucleation) were 0.00 (0.00, 0.20) and 0.06 (0.00, 16.93), respectively.

**Figure 5:**
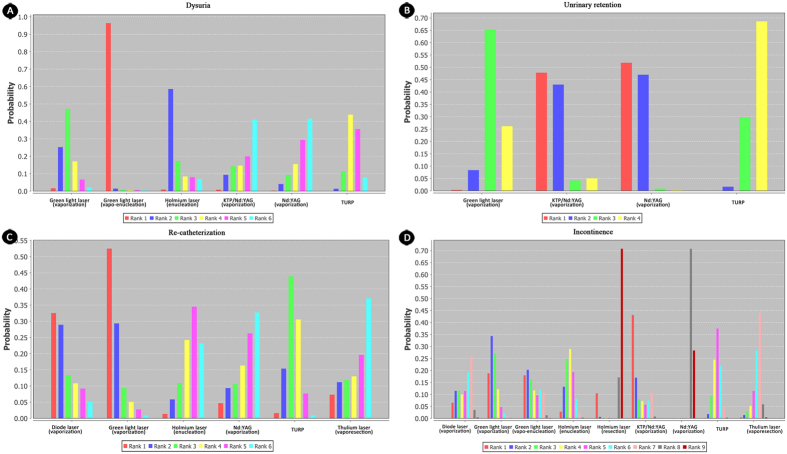
Rank of probability for short-term complicationsrelated tovoiding symptoms. (**A**) rank probability of dysuria; (**B**) rank probability of urinary retention; (**C**) rank probability of re-catheterization; (**D**) rank probability of incontinence.Thulium laser was absentfor dysuria and urinary retension. Nd:YAG/KTP was not available for re-catheterization.

The Nd:YAG laser through vaporization technique was the most common technique inducing urinary retention. Besides, re-catheterization was always seen in patients underwent green light laser (vaporization) and diode laser (vaporization); and it was barely occurred in Nd:YAG laser (vaporization), basically corresponding with the outcome of dysuria but not urinary retention (Fig. 5A–C). KTP/Nd:YAG (vaporization) and green light (vaporization) were mostly related with post-operation incontinence(Fig. 5D).

**Figure 6:**
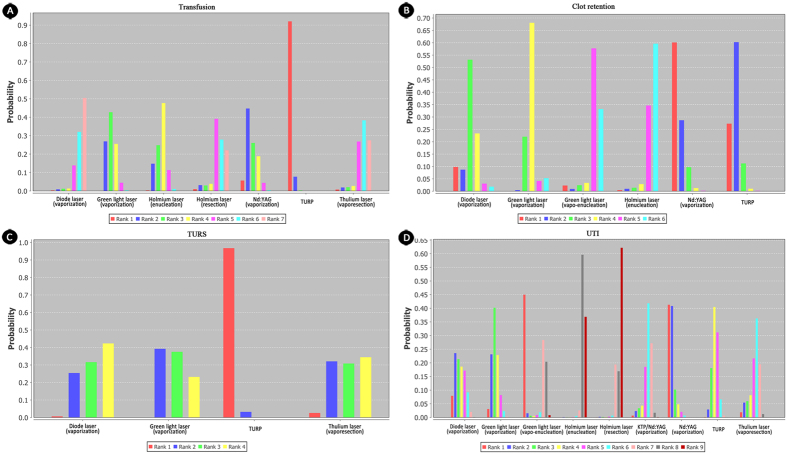
Rank of probability for other short-term complications-related outcomes. (**A**) rank probability of transfusion; (**B**) rank probability of clot retention; (**C**) rank probability of TURS; (**D**) rank probability of UTI. Thulium laser was absent for clot retension andNd:YAG/KTP was not available for clot retention, transfusion and TURS. Holmium laser was not included in TURS outcome.

Transfusion was mostly seen in TURP but rarely in diode laser(vaporization) and thulium laser (vapo–resection); Nd:YAG laser (vaporization) and TURP had much higher rates of occurrence of clot retention, while holmium laser (enucleation) had the least rate(Fig. 6A,B). In addition, TURS was most seen in patients treated with TURP (Fig. 6C). While considering UTI, the most related technique was Nd:YAG laser through vaporization (Fig. 6D).

### Long-term and other complications-related outcomes

**Figure 7:**
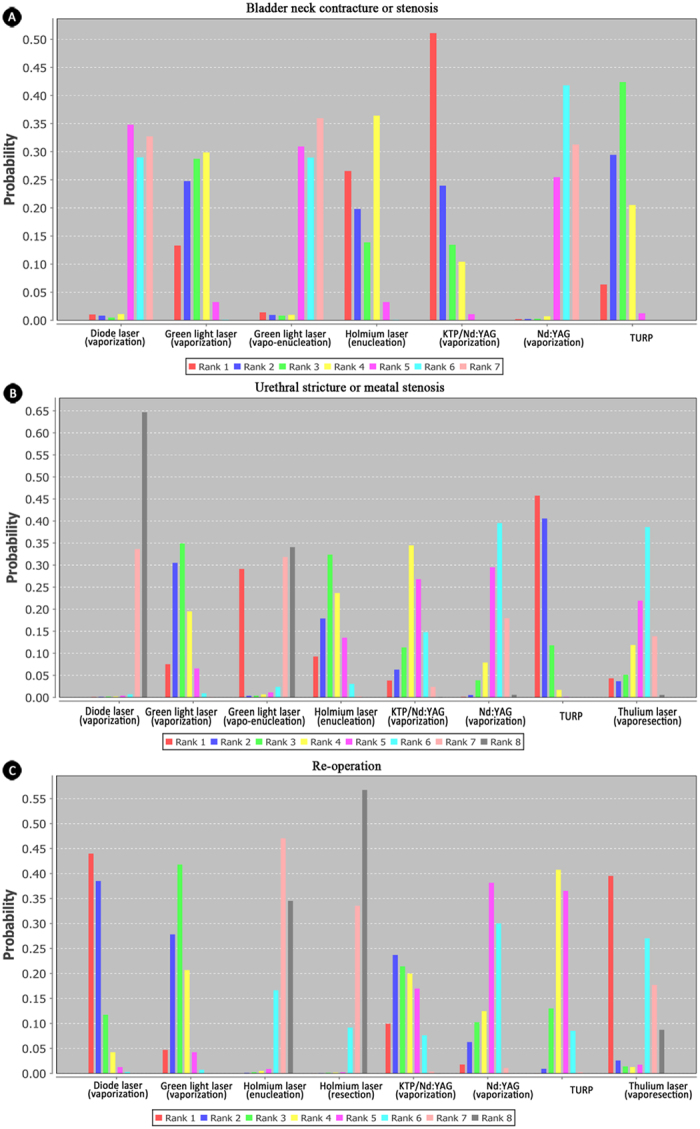
Rank of probability for long-term and other complications-related outcomes. (**A**) rank probability of bladder neck contracture or meatal stenosis; (**B**) rank probability of urethral stricture or meatal stenosis; (**C**) rank probability of re-operation. Thulium laser was not availably compared for outcome bladder neck contracture or meatal stenosis. All six kinds of lasers through different surgical techniquesand TURP were availably compared in the network meta-analysis of outcomes urethral stricture or meatal stenosis and re-operation.

Long-term complications-related outcomes, including bladder neck contracture or stenosis and urethral stricture or meatal stenosis, were analyzed in our network meta-analysis. Bladder neck contracture or stenosis was frequently occurred in KTP/Nd:YAG (vaporization) and holmium laser (enucleation); while green light (vaporization) and diode laser (vaporization)were on the contrary (Fig. 7A). The relative effect estimate of green light (vaporization) versus KTP/Nd:YAG (vaporization) was 0.47 (0.02, 5.05). Intriguingly, green light through vaporization plus enucleation had fewer bladder neck contracture or stenosis than vaporization alone. TURP was mostly associated with urethral stricture or meatal stenosis, and diode laser (vaporization) had the least incidence (Fig. 7B). Analysis of green light through different techniques showed that combination of vaporization and enucleation had less urethral stricture or meatal stenosis, compared to vaporization alone.

Re-operation was analyzed as other complications-related outcomes and was reported in 13 studies^5^,^9^,^11^,^16^,^18^,^21^,^22^,^28^,^32^,^33^,^34^,^35^,^36^,^38^,^41^. However, the reasons for re-operation were not adequately interpreted in all included studies. Therefore, it was difficult to split it into subgroup analysis, and network meta-analysis was conducted for the overall re-operation rate, which was mostly seen in diode laser (vaporization) and thulium laser (vapo-resection) (Fig. 7C).

## Discussion

TURP is always considered as the gold standard surgical treatment for patients with BPH; however, it is still associated with significant morbidity and mortality, such as TURS and transfusion. In the past two decades, both researchers and surgeons devoted to developing novel surgical treatments for pursuing a much better efficacy, as well as much more improvement of safety. Since Nd:YAG laser was firstly reported being used in the treatment of BPH by Costello and colleagues in 1992^43^, more lasers were introduced to treat BPH with improved surgical safety and efficacy. Afterwards, the improvement of technology led the appearance of innovative lasers applying in surgical practice of BPH. At present, the most often used lasers are holmium laser, green light laser and thulium laser with different surgical techniques, both in clinical practice and academic research^44^.

However, among diverse kinds of laser techniques, there is no completely or absolutely perfect intervention. Each laser has its advantages or disadvantages with different clinical outcomes. How to select the best surgical treatment for BPH is difficult but be of importance. Thus, it is worthy of comparing different surgical techniques, no matter direct or indirect studies. And to our knowledge, this is the first study, applying Bayesian analytical method of network meta-analysis, to indirectly compare the efficacy and safety of different lasers with TURP in the treatment of BPH.

Based on our network meta-analysis, holmium laser achieved a better Q max and less PVR, and thulium laser achieved a lower IPSS than other lasers with different techniques and TURP. In the aspect of peri-operation-related outcomes, holmium laser through resection technique took the longest operating time and thulium laser (vapo-resection) ranked in the middle. It was also proven by a direct analysis that thulium laser required a longer operating time than holmium laser (72.4 vs 61.5 minutes, p = 0.034)^42^. However, both the two techniques had shorter time of indwelling catheter and time of stay in hospital resulting in a reduced incidence of complications. So, holmium laser and thulium laser showed a better surgical efficacy and had a higher safety with the least incidence of complications.

The resection and enucleation techniques of thulium and holmium lasers were seen as similar as open prostatectomy in the complete removal of the prostatic lobes^31^,^39^,^42^. The thulium laser could perform a smooth incision or vaporization by continuous mode^42^. This might decrease the occurrence of LUTS and improve IPSS after operation. The holmium laser always was characterized as scar-free disruption and its incision could reach the surgical capsule of prostate^31^,^39^. And this might increase the outflow of bladder and reduce the PVR. But all these advantages were still not enough to explain the better effectiveness of holmium laser and thulium laser techniques in improving patients’ obstructive symptoms.

For peri-operation-related outcomes and the incidences of complications, green light laser through vapo-enucleation was more frequent associated with dysuria. Urinary retention was frequently seen in Nd:YAG laser alone or combined with KTP technique. Also, re-placement of catheter was mainly associated with green light laser and diode laser techniques. It was very likely that these two techniques could easily induce tissue edema and cause deficient effect when resecting the apex of prostate. Nd:YAG/KTP, diode laser, green light laser and TURP were observed with more complications (particularly green light laser) only with medium efficacies.

As shown in the analysis, we compared six kinds of laser techniques and TURP. Nowadays, since the numerous postoperative complications of the initial laser procedures and the improvement of new equipment, some lasers had to be eliminated. Nd:YAG laser technique was firstly applied in the surgical treatment of BPH and is now abandoned in clinical practice, for its incapability in ablating prostate tissue immediately, postoperative frequency and urgency led by deep tissue necrosis and high rate of re-operation^45^. As show in the study, Nd:YAG technique achieved the worst surgical effect. In contrast to obsolete laser procedures, the current higher powered lasers—holmium laser, thulium laser, green light laser and diode laser—are able to ablate prostatic tissue rapidly. Holmium laser is optimized for incision, and green light laser is optimized for vaporization. Previously researches showed that these two techniques had the superiorities over other lasers in the respect of functional outcomes, which were inconsistent with the results of our analysis^44^.

According to the results of our network meta-analysis, it seemed that the choice of laser techniques for BPH should be depended on what kinds of aims the patient wanted to get benefit, such as TURP may not be the best choice for patients with previous documented or suspected urethral stricture or meatal stenosis, as its incidence of postoperative urethral stricture or meatal stenosis was the highest.

There were some limitations in this network meta-analysis, such as included RCTs studied from 1995 to 2015, evident differences in sample sizes and significant transformation in techniques of the same lasers (green light laser improved form initial 80-W to 120-W, and subsequently to the current laser at 180-W)^5^,^7^,^8^,^9^,^10^,^13^,^18^,^22^,^24^,^25^,^26^,^35^,^40^. Although all authors were contacted to provide un-reported data for the integrity of analysis, the results of this study were restricted by incomplete data reported by included studies. Also, time-points of analyzed outcome measures were restrained to 12 months, for the limited data reported. But this review still enjoyed several advantages—rigidly analytical process by pre-published protocol, analyses of both lasers and TURP, and indirect comparisons in predicting efficacies of one technique to another due to the absence of head-to-head RCTs.

Furthermore, it might be encouraged for us to offer referable ideas for future researches and valuable advice for clinical surgeons by using this first comprehensive systematic review and network meta-analysis to assess the efficacy and safety of different laser techniques and TURP for BPH.

In conclusion, this is the first time to indirectly compare the efficacy and safety of different lasers with TURP for BPH by applying network meta-analysis. To date, no completely or absolutely perfect laser technique could be found to take the place of TURP in the surgical treatment of BPH. Holmium laser and thulium laser may seem to be relatively better in terms of surgical efficacy and safety, so that these two lasers might be preferred in the selection of optimal laser surgery. Actually, much longer-term, larger-scale and higher quality head-to-head RCTs are needed to validate the conclusions.

## Methods

### Protocol and registration

We developed a protocol defining the search strategy and a systematic review was performed to identify those randomized controlled trials (RCTs) investigating the efficacy and safety of different lasers or TURP for BPH. The review was registered on PROSPERO of the Centre for Reviews and Dissemination (CRD) (CRD42015024227). The data searching, study selection, quality assessment of included studies and data extraction were performed independently by two researchers (Z.X.M. and S.P.F.). Disagreements were resolved by discussing or with the help of a third investigator to reach the final consensus.

### Eligibility and exclusion criteria

The eligibility criteria included: 1) Patients with BPH needed surgery intervention; 2) RCTs comparing different lasers and TURP (comparing different laser with one another), either laser alone or in combination with others, for the treatment of BPH; 3) Each laser may be through different surgical techniques:vaporization, resection of tissue pieces, enucleation, or combinations; 4) Published in English, German, French, Italian, Russian, Dutch, and Japanese.

Exclusion criteria: 1) Patients with neurogenic bladder disorder, urethral strictures, history of prostate adenocarcinoma or any previous prostatic, and bladder neck or urethral surgery. 2) Non-RCTs, reviews, reports only focusing on laboratory findings, trials published only as abstracts. 3) Not published in English, German, French, Italian, Russian, Dutch, and Japanese.

Outcome measures included: 1) Effectiveness-related outcomes: maximum flow rate (Q max), International Prostate Symptom Score (IPSS), post-void residual volume (PVR); 2) Perioperation-related outcomes: operating time, hospitalization and catheterization; 3) Complications-related outcomes: ① short-term complications-related outcomes (always occurred within one month after operation): dysuria, re-catherization, urine retention, clot retention, transfusion, transurethral resection syndrome (TURS), urine incontinence, and urinary tract infection (UTI); ② long-term complications-related outcomes (always occurred after one month of operation): bladder neck contracture or stenosis, urethral stricture or meatal stenosis; ③ other complication-related outcome: re-operation because of any other complications.

### Data resources and searches

MEDLINE (1966–2015.5) and EMBASE (1947–2015.5) were searched. Further searches were undertaken in the Cochrane Central Register of Controlled Trials (CENTRAL) (1948–2015.5), WHO International Clinical Trial Registration Platform (ICTRP) (2004–2015.5), and Clinical Trial.gov (1999–2015.5). The following terms and keywords were used: laser, transurethral resection of prostate, TURP, benign prostatic hyperplasia, and BPH. Included trials’ references were searched for more studies and experts in the field were consulted.

### Study selection and data extraction

Two investigators independently assessed the titles and abstracts of the searched results. The full text versions of those studies, which were potentially eligible, were then assessed. For the study design, selection criteria, participant’s characteristics, interventions, outcome measures, study duration, results and other data of each included study were extracted. Extracted contents were recorded on data extraction forms, which were designed according to the advice given in the Cochrane Handbook^46^. We contacted authors to seek additional information where data were not reported or not clear.

### Assessment of risk

Two independent reviewers (Z.X.M. and S.P.F.) used RevMan 5 software to assess the risk of bias of all included studies according to the Cochrane Handbook^47^, as follows: 1). adequate sequence generation; 2). allocation concealment; 3). incomplete outcome data; 4). free of selective reporting; 5). free of other bias. The judgments were categorized as ‘yes’ (low risk’ of bias), ‘no’ (high risk of bias) or ‘unclear’ (unclear risk of bias).

### Data synthesis

ADDIS software (version 1.16.6) was applied to conduct indirect comparison analysis (network meta-analysis to compare different interventions not directly matched). Network meta-analysis led us to predict the likely comparable estimates between indirect comparisons based on two or more studies with one common intervention. Using Bayesian approach, the relative effect estimates were calculated and could be used to estimate the probability that which one was the best. Node-split analysis was utilized to check inconsistency among comparisons, and p < 0.05 was set as significant inconsistency.

## Additional Information

**How to cite this article**: Zhang, X. *et al*. Different lasers in the treatment of benign prostatic hyperplasia: a network meta-analysis. *Sci. Rep.* **6**, 23503; doi: 10.1038/srep23503 (2016).

## Figures and Tables

**Table 1:** Characteristics of included studies.

**Studies**	**Interventions**	**N**	**Baseline characteristics**					
			**Age (years)**	**Surgical techniques of Lasers**	**Q max (ml/s)**	**IPSS score**	**PVR (ml)**	**Prostate volume (cc)**
Ahyai2007 + Kuntz 2004	Holmium laser vs TURP	200	68.3 ± 91.8	E	5.4 ± 30.6	NA	226 ± 2423.5	51.7 ± 201.5
Al-Ansari 2010	Green light vs TURP	120	66.7 ± 8.7	V	6.7 ± 2.1	27.6 ± 2.5	55.1 ± 23.1	61.1 ± 21.0
Anson 1995	Nd:YAG vs TURP	151	68.1 ± 7.5	V	9.7 ± 3.8	NA	116.8 ± 104.2	76.2 ± 11.3*
Bachmann 2014	Green light vs TURP	269	65.7 ± 6.7	V	9.7 ± 3.3	21.4 ± 6.1	110.0 ± 96.2	47.4 ± 19.2
Bouchier-Hayes 2006	Green light vs TURP	76	65.7 ± 43.1	V	NA	38.7 ± 93.1	65.7 ± 43.1	NA
Bouchier-Hayes 2010	Green light vs TURP	119	65.7 ± 54.2	V	8.8 ± 2.8	25.3 ± 5.8	65.7 ± 54.2	8.8 ± 2.8
Capita´n 2011	Green light vs TURP	100	68.7 ± 7.7	V	6.0 ± 3.6	23.6 ± 4.8	68.7 ± 7.7	6.0 ± 3.6
Carter 1999	KTP/Nd:YAG vs TURP	204	67.4 ± 7.6	V	9.4 ± 3.0	NA	67.4 ± 7.6	9.4 ± 3.0
Cowles 1995	Nd:YAG vs TURP	115	66.4 ± 7.3	V	NA	19.8 ± 5.5	66.4 ± 7.3	NA
Elshal 2015	Green light vsHolmium laser	103	72.6 ± 9.1	V + Evs E	7.8 ± 2.3	22.7 ± 5.2	72.6 ± 9.1	7.8 ± 2.3
Eltabey 2010	Holmium laser vs TURP	80	67.9 ± 8.6	E	8.3 ± 2.5	24.0 ± 4.5	67.9 ± 8.6	8.3 ± 2.5
Fraundorfer 2001	Holmium laser vs TURP	120	NA	R	9.0 ± 3.1	NA	NA	9.0 ± 3.1
Gilling 1998	Nd:YAGvs Holmium laser	44	66 ± 42.7	R vs V	NA	NA	155.0 ± 158.5	45.5 ± 64.0
Gupta 2006	Holmium laser vs TURP	100	66.5 ± 9.1	E	4.8 ± 4.2	23.9 ± 4.1	66.5 ± 9.1	4.8 ± 4.2
Horasanli 2007	Green light vs TURP	76	68.8 ± 6.9	V	8.9 ± 5.4	19.5 ± 6.0	68.8 ± 6.9	8.9 ± 5.4
Keoghane 1996 + 2000	Nd:YAG vs TURP	87	69.8^#^	V	11.6 ± 4.7	NA	NA	53.0 ± 25.1
Kursh 2003	Diode laser vs TURP	72	68.5 ± 46.5	V	NA	NA	68.5 ± 46.5	NA
Liatsikos 2012	Green light vs TURP	60	68.8 ± 7.9	V	NA	NA	68.8 ± 7.9	NA
Liedberg 2003	Nd:YAG vs TURP	31	NA	V	8.0 ± 3.0	18.3 ± 8.0	NA	8.0 ± 3.0
Lukacs 2012	Green light vs TURP	136	67.3 ± 7.7	V	7.8 ± 2.7	21.0 ± 2.4	67.3 ± 7.7	7.8 ± 2.7
Mohanty 2012	Green light vs TURP	117	66.2 ± 8.8	V	7.1 ± 1.9	20.4 ± 3.6	66.2 ± 8.8	7.1 ± 1.9
Pereira-Correia 2012	Green light vs TURP	20	65.3 ± 43.9	V	8.7 ± 13.4	22.8 ± 36.3	65.3 ± 43.9	8.7 ± 13.4
Razzaghi 2007	Nd:YAG vs TURP	87	66.6 ± 8.2	V	6.1 ± 5.6	NA	165.0 ± 116.2	35.6 ± 8.2*
Razzaghi 2014	Diode laser vs TURP	115	68.3 ± 8.3	V	6.5 ± 2.1	24.1 ± 6.8	59.4 ± 61.3	60.3 ± 15.1
Rigatti 2005	Holmium laser vs TURP	100	64.8 ± 6.9	E	8.0 ± 3.4	21.7 ± 6.9	NA	58.3 ± 29.6
Shingleton 1999	KTP/Nd:YAG vs TURP	100	67.8 ± 7.6	V	7.5 ± 3.7	NA	NA	30.9 ± 18.6
Sun 2014	Holmium laser vs TURP	164	72.0 ± 7.5	E	5.5 ± 1.7	24.5 ± 3.8	111.9 ± 109.1	NA
Tan2003 + Wilson 2006 + Gilling 2012	Holmium laser vs TURP	61	71.0 ± 5.8	E	8.4 ± 2.5	NA	NA	73.8 ± 29.3
Telli 2015	Green light vs TURP	101	68.2 ± 60.2	V	11.8 ± 30.6	19.4 ± 37.4	63.1 ± 383.5	56.9 ± 60.4
Tuhkanen 1999-a	Nd:YAG vs TURP	45	67.0 ± 34.4	V	7.8 ± 15.4	NA	135.1 ± 491.4	55.0 ± 57.8
Tuhkanen 1999-b	Nd:YAG vs TURP	50	67.0 ± 25.9	V	8.6 ± 3.5	NA	116.5 ± 88.6	27 ± 31.6
Van Melick 2003	Nd:YAG vs TURP	95	66.5 ± 8.5	V	11.5 ± 4.0	17.8 ± 6.4	NA	37.0 ± 11.0
Xia 2007	Thulium laser vs TURP	100	69.7 ± 7.5	V + R	8.1 ± 2.9	21.4 ± 6.3	89.2 ± 34.4	NA
Xue 2013	Green light vs TURP	200	71.6 ± 11.0	V	8.1 ± 3.7	23.1 ± 5.0	149.7 ± 103.1	66.6 ± 24.1
Yan 2013	Thulium laser vs TURP	80	73.5 ± 7.3	V + R	7.7 ± 2.7	22.2 ± 4.9	74.4 ± 35.1	NA
Zhang 2011	Holmium laser vsThulium laser	133	74.2 ± 9.8	E vs E	7.0 ± 3.7	24.1 ± 3.0	64.6 ± 33.0	44.7 ± 23.6

Age, Q max, IPSS, PVR and Prostate volume-mean ± SD. *Prostate volume-gram; ^#^data only reported mean, no SD or other kind of data; NA-data not available; E-Enucleation; V-Vaporization; R-Resection.

## References

[b1] Wei J. T., Calhoun E. & Jacobsen S. J. Urologic diseases in America project: benign prostatic hyperplasia. J Urol. 173, 1256–61(2005). 15758764 10.1097/01.ju.0000155709.37840.fe

[b2] Gravas S.. Management of Non-Neurogenic Male Lower Urinary Tract Symptoms (LUTS), incl. Benign Prostatic Obstruction (BPO). EAU guidelines. 13–45 (2015). 10.1016/j.eururo.2014.12.038 25613154

[b3] Anson K. . A multicenter, randomized, prospective study of endoscopic laser ablation versus transurethral resection of the prostate. Urology. 46, 305–10 (1995). 7544932 10.1016/S0090-4295(99)80211-8

[b4] Liedberg F. . Interstitial laser coagulation versus transurethral resection of the prostate for benign prostatic enlargement–a prospective randomized study. Scand J Urol Nephrol. 37, 494–7 (2003). 14675923 10.1080/00365590310001773

[b5] Al-Ansari A. . GreenLight HPS 120-W laser vaporization versus transurethral resection of the prostate for treatment of benign prostatic hyperplasia: a randomized clinical trial with midterm follow-up. Eur Uro. 58, 349–55 (2010). 10.1016/j.eururo.2010.05.026 20605316

[b6] Ahyai S. A., Lehrich K. & Kuntz R. M. Holmium laser enucleation versus transurethral resection of the prostate: 3-year follow-up results of a randomized clinical trial. Eur Urol. 52, 1456–63 (2007). 17499427 10.1016/j.eururo.2007.04.053

[b7] Bachmann A. . 180-W XPS GreenLight laser vaporisation versus transurethral resection of the prostate for the treatment of benign prostatic obstruction: 6-month safety and efficacy results of a European MulticentreRandomised Trial–the GOLIATH study. Eur Urol. 65, 931–42(2014). 24331152 10.1016/j.eururo.2013.10.040

[b8] Bouchier-Hayes D. M. . KTP laser versus transurethral resection: early results of a randomized trial. J Endourol. 20, 580–5 (2006). 16903819 10.1089/end.2006.20.580

[b9] Bouchier-Hayes D. M. . A randomized trial of photoselective vaporization of the prostate using the 80-W potassium-titanyl-phosphate laser vs transurethral prostatectomy, with a 1-year follow-up. BJU Int. 105, 964–9 (2010). 19912196 10.1111/j.1464-410X.2009.08961.x

[b10] Capitan C. . GreenLight HPS 120-W laser vaporization versus transurethral resection of the prostate for the treatment of lower urinary tract symptoms due to benign prostatic hyperplasia: a randomized clinical trial with 2-year follow-up. Eur Urol. 60, 734–9 (2011). 21658839 10.1016/j.eururo.2011.05.043

[b11] Carter A. . A prospective randomized controlled trial of hybrid laser treatment or transurethral resection of the prostate, with a 1-year follow-up. BJU Int. 83, 254–9 (1999). 10233489 10.1046/j.1464-410x.1999.00936.x

[b12] Cowles R. S. 3rd. . A prospective randomized comparison of transurethral resection to visual laser ablation of the prostate for the treatment of benign prostatic hyperplasia. Urology. 46, 155–60(1995). 7542818 10.1016/s0090-4295(99)80185-x

[b13] Ahmed M. E., Mohamed A. E. & Ahmed R. E. GreenLight^TM^ Laser (XPS) PhotoselectiveVapo-Enucleation versus Holmium Laser Enucleation of the Prostate for the Treatment of Symptomatic Benign Prostatic Hyperplasia: A Randomized Controlled Study. J Urol. 193, 927–34 (2015). 25261801 10.1016/j.juro.2014.09.097

[b14] Eltabey M. A., Sherif H. & Hussein A. A. Holmium laser enucleation versus transurethral resection of the prostate. Can J Urol. 17, 5447–52 (2010). 21172109

[b15] Fraundorfer M. R. . Holmium laser resection of the prostate is more cost effective than transurethral resection of the prostate: results of a randomized prospective study. Urology. 57, 454–8 (2001). 11248619 10.1016/s0090-4295(00)00987-0

[b16] Gilling P. J. . Holmium laser resection of the prostate versus neodymium:yttrium-aluminum-garnet visual laser ablation of the prostate: a randomized prospective comparison of two techniques for laser prostatectomy. Urology. 51, 573–7 (1998). 9586609 10.1016/s0090-4295(97)00642-0

[b17] Gupta. N. . Comparison of standard transurethral resection, transurethral vapour resection and holmium laser enucleation of the prostate for managing benign prostatic hyperplasia of >40 g. BJU Int. 97, 85–9 (2006). 16336334 10.1111/j.1464-410X.2006.05862.x

[b18] Horasanli. K. . Photoselective potassium titanyl phosphate (KTP) laser vaporization versus transurethral resection of the prostate for prostates larger than 70 mL: a short-term prospective randomized trial. Urology. 71, 247–51 (2008). 18308094 10.1016/j.urology.2007.09.017

[b19] Keoghane S. R. . The Oxford Laser Prostate Trial: a double-blind randomized controlled trial of contact vaporization of the prostate against transurethral resection; preliminary results. Br J Urol. 77, 382–5 (1996). 8814842 10.1046/j.1464-410x.1996.98310.x

[b20] Keoghane S. R. . A double-blind randomized controlled trial and economic evaluation of transurethral resection vs contact laser vaporization for benign prostatic enlargement: a 3-year follow-up. BJU Int. 85, 74–8 (2000). 10619950 10.1046/j.1464-410x.2000.00407.x

[b21] Kursh E. D. . Interstitial laser coagulation versus transurethral prostate resection for treating benign prostatic obstruction: a randomized trial with 2-year follow-up. Urology. 61, 573–8 (2003). 12639650 10.1016/s0090-4295(02)02420-2

[b22] Liatsikos. E. . PhotoselectiveGreenLight laser vaporization versus transurethral resection of the prostate in Greece: a comparative cost analysis. J Endourol. 26, 168–73 (2012). 22050499 10.1089/end.2011.0089

[b23] Kuntz R. M. . Transurethral holmium laser enucleation of the prostate versus transurethral electrocautery resection of the prostate: a randomized prospective trial in 200 patients. J Urol. 172, 1012–6 (2004). 15311026 10.1097/01.ju.0000136218.11998.9e

[b24] Lukacs B. . Photoselective vaporization of the prostate with GreenLight 120-W laser compared with monopolar transurethral resection of the prostate: a multicenter randomized controlled trial. Eur Urol. 61, 1165–73 (2012). 22341632 10.1016/j.eururo.2012.01.052

[b25] Mohanty N. K. . Photoselective vaporization of prostate vs. transurethral resection of prostate: A prospective, randomized study with one year follow-up. Indian J Urol. 28, 307–12 (2012). 23204660 10.4103/0970-1591.102708 PMC3507401

[b26] Pereira-Correia J. A. . GreenLight HPS 120-W laser vaporization vs transurethral resection of the prostate (<60 ml): a 2-year randomized double-blind prospective urodynamic investigation. BJU Int. 110, 1184–9 (2012). 22257240 10.1111/j.1464-410X.2011.10878.x

[b27] Razzaghi M. R. . Laser prostatectomy versus transurethral resection of prostate in the treatment of benign prostatic hyperplasia. Saudi Med J. 28, 68–72 (2007). 17206293

[b28] Razzaghi M. R. . Diode laser (980 nm) vaporization in comparison with transurethral resection of the prostate for benign prostatic hyperplasia: randomized clinical trial with 2-year follow-up. Urology. 84, 526–32 (2014). 25168526 10.1016/j.urology.2014.05.027

[b29] Rigatti L., Naspro R., Salonia A. . Urodynamics after TURP and HoLEP in urodynamically obstructed patients: are there any differences at 1 year of follow-up? Urology. 67, 1193–8 (2006). 16750253 10.1016/j.urology.2005.12.036

[b30] Shingleton W. B. . A randomized prospective study of laser ablation of the prostate versus transurethral resection of the prostate in men with benign prostatic hyperplasia. Urology. 54, 1017–21(1999). 10604701 10.1016/s0090-4295(99)00319-2

[b31] Sun N. . Holmium laser enucleation of the prostate versus transurethral resection of the prostate: a randomized clinical trial. Int Urol Nephrol. 46, 1277–82 (2014). 24492988 10.1007/s11255-014-0646-9

[b32] Tan A. H. . A randomized trial comparing holmium laser enucleation of the prostate with transurethral resection of the prostate for the treatment of bladder outlet obstruction secondary to benign prostatic hyperplasia in large glands (40 to 200 grams). J Urol. 170, 1270–4 (2003). 14501739 10.1097/01.ju.0000086948.55973.00

[b33] Wilson L. C. . A randomised trial comparing holmium laser enucleation versus transurethral resection in the treatment of prostates larger than 40 grams: results at 2 years. Eur Urol. 50, 569–73 (2006). 16704894 10.1016/j.eururo.2006.04.002

[b34] Gilling P. J. . Long-term results of a randomized trial comparing holmium laser enucleation of the prostate and transurethral resection of the prostate: results at 7 years. BJU Int. 109, 408–11 (2012). 21883820 10.1111/j.1464-410X.2011.10359.x

[b35] Telli O. . A prospective, randomized comparative study of monopolartransurethral resection of the prostate versus photoselective vaporization of the prostate with GreenLight 120-W laser, in prostates less than 80 cc. Ther Adv Urol. 7, 3–8 (2015). 25642290 10.1177/1756287214556643 PMC4294803

[b36] Tuhkanen K., Heino A. & Alaopas M. Hybrid laser treatment compared with transurethral resection of the prostate for symptomatic bladder outlet obstruction caused by a large benign prostate: a prospective, randomized trial with a 6-month follow-up. BJU Int. 84, 805–9 (1999). 10532976 10.1046/j.1464-410x.1999.00316.x

[b37] Tuhkanen K., Heino A. & Ala-Opas M. Contact laser prostatectomy compared to TURP in prostatic hyperplasia smaller than 40 ml. Six-month follow-up with complex urodynamic assessment. Scand J Urol Nephrol. 33, 31–4 (1999). 10100361 10.1080/003655999750016249

[b38] van Melick H. H. . A randomized controlled trial comparing transurethral resection of the prostate, contact laser prostatectomy and electrovaporization in men with benign prostatic hyperplasia: analysis of subjective changes, morbidity and mortality. J Urol. 169, 1411–6 (2003). 12629374 10.1097/01.ju.0000054657.59200.97

[b39] Xia S. J. . Thulium laser versus standard transurethral resection of the prostate: a randomized prospective trial. Eur Urol. 53, 382–89(2008). 17566639 10.1016/j.eururo.2007.05.019

[b40] Xue B. . GreenLight HPS 120-W laser vaporization versus transurethral resection of the prostate for treatment of benign prostatic hyperplasia: a prospective randomized trial. J Xray Sci Technol. 21, 125–32 (2013). 23507858 10.3233/XST-130359

[b41] Yan H. . Thulium laser vaporesection versus standard transurethral resection of the prostate: a randomized trial with transpulmonarythermodilution hemodynamic monitoring. Int J Urol. 20, 507–12 (2013). 23088252 10.1111/j.1442-2042.2012.03183.x

[b42] Zhang F. . Thulium laser versus holmium laser transurethral enucleation of the prostate: 18-month follow-up data of a single center. Urology. 79, 869–74 (2012). 22342411 10.1016/j.urology.2011.12.018

[b43] Costello A. J., Johnson D. E. & Bolton D. M. Nd:YAG laser ablation of the prostate as a treatment for benign prostatic hypertrophy. Lasers Surg Med. 12, 121–4 (1992). 1374142 10.1002/lsm.1900120202

[b44] Rieken M. & Bachmann A. Laser treatment of benign prostate enlargement—which laser for which prostate? Nat Rev Urol. 11, 142–52 (2014). 24595121 10.1038/nrurol.2014.23

[b45] Kramer M. W. . Current evidence for transurethral laser therapy of non-muscle invasive bladder cancer. World J Urol. 29, 433–42 (2011). 21544662 10.1007/s00345-011-0680-5

[b46] Higgins J. P. T. & Deeks J. J. Chapter 7: Selecting studies and collecting data. In: Higgins J. P. T., Green S. (editors), Cochrane Handbook for Systematic Reviews of Interventions. Version 5.1.0 [updated March 2011]. The Cochrane Collaboration 2011. Available at www.cochrane-handbook.org.

[b47] Schünemann H. J. O. A. . Chapter 12: Interpreting results and drawing conclusions. In: Higgins J. P. T., Green S. (editors), Cochrane Handbook for Systematic Reviews of Interventions. Version 5.1.0 [updated March 2011]. The Cochrane Collaboration 2011. Available at www.cochranehandbook.org.

